# The incidences and risk factors related to early dysphagia after anterior cervical spine surgery: A prospective study

**DOI:** 10.1371/journal.pone.0173364

**Published:** 2017-03-07

**Authors:** Jia-Ming Liu, Wei-Lai Tong, Xuan-Yin Chen, Yang Zhou, Wen-Zhao Chen, Shan-Hu Huang, Zhi-Li Liu

**Affiliations:** Department of Orthopaedic Surgery, the First Affiliated Hospital of Nanchang University, Nanchang, PR. China; Universita degli Studi di Palermo, ITALY

## Abstract

Dysphagia is a common complication following anterior cervical spine surgery (ACSS). The incidences of dysphagia were variable and controversial. The purpose of this study was to determine the incidence of early dysphagia after ACSS with a new scoring system, and to identify the risk factors of it. A prospective study was carried out and patients who underwent ACSS from March 2014 to August 2014 in our hospital were included in this study. A self-designed dysphagia questionnaire was delivered to all of the patients from the first day to the fifth day after ACSS. Perioperative characteristics of patients were recorded, and incidences and risk factors of dysphagia were analyzed. A total of 104 patients who underwent ACSS were included and incidences of dysphagia from the first to the fifth day after ACSS was 87.5%, 79.81%, 62.14%, 50% and 44.23%, respectively. There was a good correlation between the new dysphagia scoring system and Bazaz scoring system (*P* < 0.001). Operative time and body mass index (BMI) were the risk factors for dysphagia during the first to the second day postoperatively. However, the dC2-C7angle was the main risk factor for dysphagia from the third to the fifth day after surgery. There were comparatively high incidences of early dysphagia after ACSS, which may be ascribed to operative time, BMI and the dC2-C7 angle.

## Introduction

Anterior cervical spine surgery (ACSS) is commonly used for many different kinds of spine conditions, such as trauma and degenerative cervical disease [1–3]. The anterior approach is easy to relieve pain and recover the function of patients. However, a variety of complications related to ACSS have been reported. One of the most common complications is dysphagia [4–6]. Although dysphagia is a transient problem for patient undergoing ACSS, it will affect patient's function recovery and decreases the postoperative quality of life.

The incidences of dysphagia after ACSS were reported from 1.7% to 71% [7], which were variable and controversial. One of the reasons for this, is the different scoring systems used for dysphagia measurement. Although many scales have been reported for dysphagia assessment, the most popular one is Bazaz dysphagia scoring system [8], which defined dysphagia as four grades: none, mild, moderate and severe. However, the definition of Bazaz scale is simple and just focuses on the swallowing difficulty of patients, which will underestimate the incidence of dysphagia. Thus, we designed a new scoring system to evaluate the early dysphagia of patients undergoing ACSS.

The purpose of this study is to determine the incidences of early dysphagia by a new scoring system and identify the risk factors related to dysphagia after ACSS.

## Materials and methods

This study was approved by the ethics board committee of the First Affiliated Hospital of Nanchang University, and written informed consents were received from all the participants. A prospective study was performed and patients who underwent ACSS between March 2014 and August 2014 were included in this study. The inclusion criteria for the study were patients diagnosed with one or two-level adjacent cervical spondylotic myelopathy and underwent anterior cervical discectomy and fusion (ACDF) or anterior cervical corpectomy and fusion (ACCF). Patients who had the history of cervical spine surgery, with dysphagia before surgery, lost during follow-up period, and with incomplete medical data were excluded from the study.

All the surgeries were performed by two senior surgeons in our hospital. After general anesthesia, a satisfactory endotracheal intubation was conducted. The surgery was begun with a right-sided incision. Deep retractors were used to provide a relatively capacious exposure of the operative site. All of the patients were underwent ACDF or ACCF. A closed suction drainage was routinely performed after the surgery. The drainage was removed within 72 hours when drainage volume was less than 50 ml/24h.

In order to evaluate the early dysphagia, a new scoring system was designed. Due to most of the patients undergoing ACSS complicated with dysphagia because of the pain located at the throat and the sensation of foreign body when swallowing, we chose the symptoms of pharyngeal pain and foreign body sensation as the major factors of the new scoring system. Dysphagia is divided into five grades in the new scoring system: grade I, II, III, IV and V (Table 1). Grade I dysphagia is defined as the normal one and grade V is defined as the most severe one. Early dysphagia developing during the first to the fifth day after ACSS was measured by both of self-designed dysphagia scoring system and the Bazaz scale (Table 2). Dysphagia questionnaires were sent to each patient to determine the severity of dysphagia. And the incidences of dysphagia on each day were calculated.

**Table 1:** Self-designed dysphagia scoring system.

Severity of dysphagia	Definition
Grade I	Normal
Grade II	Foreign body sensation with solids and liquids
Grade III	Foreign body sensation with liquids
Grade IV	Pain with liquids
Grade V	Pain when swallowing, that cannot eat liquids

**Table 2:** Bazaz dysphagia scoring system.

Severity of dysphagia	Definition
**None**	No episodes of difficulty swallowing
**Mild**	Only rare episodes of difficulty swallowing
**Moderate**	Occasional swallowing difficulty with solid foods
**Severe**	Swallowing difficulty with solids and liquids

In addition, all the patients' clinical characteristics, including age, gender, body mass index (BMI), operative time, blood loss, implant use, preoperative JOA score and the dC2-C7 angle were collected. The dC2-C7 angle was defined as the value that postoperative angle minus the preoperative one. The risk factors related to early dysphagia after ACSS were analyzed.

Statistical analysis was performed by SPSS 17.0 software (SPSS Inc., Chicago, Illinois). Spearman rank correlation analysis was used to determine the correlation between self-designed scoring system and the Bazaz scale. *Chi-square* test was used to defect the differences between patients with and without dysphagia. A *P* < 0.05 was considered to be significant.

## Results

A total of 104 patients were involved in this study, consisting of 70 males and 34 females with an average age of 51.8±11.0 years (ranged from 19 to 78 years). The demographics and clinical characteristics of the patients were demonstrated in Table 3. Of these patients, the most common symptom before surgery was extremities numbness and weakness (41 cases, 39.4%). And 55 (52.9%) patients diagnosed as cervical spondylotic myelopathy with single-level lesion and 49 (47.1%) patients with two-level lesions.

**Table 3:** Demographics and clinical characteristics of patients included in this study.

Patient characteristics	n (%) (N = 104)
Gender	
Male	70 (67.3)
Female	34 (32.7)
Age (years)	51.8 ± 11.0
Body mass index	22.4 ± 3.1
Preoperative symptoms	
Neck pain	2 (1.9)
Extremities weakness	2 (1.9)
Neck and extremities pain	3 (2.9)
Extremities pain and weakness	3 (2.9)
Extremities pain and numbness	6 (5.8)
Neck pain and extremities numbness	7 (6.7)
Neck pain and extremities weakness	20 (19.2)
Extremities numbness	20 (19.2)
Extremities numbness and weakness	41 (39.4)
Lesion locations	
C2/3, C3/4	1 (1.0)
C3/4	8 (7.7)
C3/4, C4/5	11 (10.6)
C4/5	15 (14.4)
C4/5, C5/6	23 (22.1)
C5/6	25 (24.0)
C5/6, C6/7	13 (12.5)
C6/7	6 (5.8)
C6/7, C7/T1	1 (1.0)
C7/T1	1 (1.0)
Surgical time (min)	134.8 ± 35.3
Intraoperative blood loss (ml)	109.5 ± 88.2

**Fig 1:**
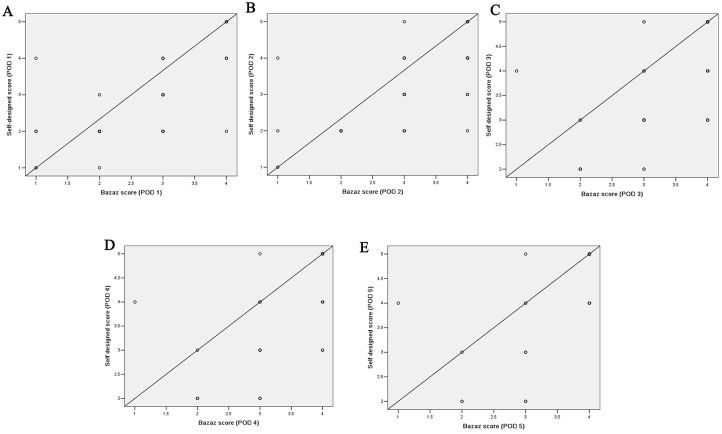
The correlation between self-designed scoring system and Bazaz scale on scatter graphs. (A) the scatter graph of the two scoring system at postoperative day 1(POD 1); (B) the scatter graph of POD 2; (C) the scatter graph of POD 3; (D) the scatter graph of POD 4; (E) the scatter graph of POD 5.

According to the self-designed dysphagia scoring system, the incidences of dysphagia from the first to the fifth day after ACSS was 87.5%, 79.81%, 62.14%, 50% and 44.23%, respectively. Due to both the variables of the two scales were ranking, Spearman rank correlation analysis was used to analyze the correlation of them. We defined the four grades of Bazaz scale (none, mild, moderate and severe) as grade 1, 2, 3 and 4, respectively. The results showed that there was a good correlation between self-designed dysphagia scoring system and Bazaz scale (Table 4 and Fig 1).

**Table 4:** The correlation between self-designed scoring system and Bazaz scoring system on dysphagia evaluation.

	POD* 1	POD* 2	POD* 3	POD* 4	POD* 5
**Correlation coefficient**	0.791	0.809	0.692	0.722	0.689
***P* value**	< 0.001	< 0.001	< 0.001	< 0.001	< 0.001

*POD: postoperative day.

Based on the statistical analysis, patients who got early dysphagia after ACSS had similar age, gender, blood loss, implant use and preoperative JOA score comparing with those without dysphagia from the first to the fifth day (*P* > 0.05) (Table 5). However, significant differences were found in operative time and BMI between patients with and without dysphagia from the first to the second day after surgery. Moreover, there was significant difference in dC2-C7 angle between patients with and without dysphagia from the third to the fifth day postoperatively. It indicated that operative time, BMI and dC2-C7 angle may be the risk factors for early dysphagia in patients undergoing ACSS.

**Table 5:** The Chi-square test results for different clinical factors between patients with and without dysphagia after anterior cervical spinal surgery.

Clinical factors	*P* value				
	POD* 1	POD* 2	POD* 3	POD* 4	POD* 5
**Age**	0.318	0.062	0.452	0.995	0.736
**Gender**	0.243	0.236	0.530	0.097	0.123
**Body mass index**	0.025	0.002	0.732	0.527	0.400
**Operating time**	0.009	0.017	0.957	0.863	0.327
**Blood loss**	0.095	0.468	0.433	0.846	0.340
**Hardware use**	0.144	0.125	0.321	0.156	0.213
**Preoperative JOA score**	0.125	0.566	0.631	0.507	0.677
**dC2-C7 angle**	0.661	0.397	0.007	0.001	0.002

*POD: postoperative day.

For the dC2-C7 angle, in order to make clear what degree was the risk factor of postoperative dysphagia, the Chi-square test was used for analysis. The results showed when the dC2-C7 angle was greater than 9°, the incidence of postoperative dysphagia was significantly increased (*P* < 0.05) (Table 6).

**Table 6:** The Chi-square test results for different degree of dC2-C7angle between patients with and without dysphagia after surgery.

dC2-C7 angle	*χ2* and *P* values		
	POD* 3	POD* 4	POD* 5
**5°**	χ2 = 0.616, P = 0.433	χ2 = 1.179, P = 0.278	χ2 = 0.015, P = 0.902
**7°**	χ2 = 0.169, P = 0.681	χ2 = 0.756, P = 0.385	χ2 = 1.277, P = 0.258
**8°**	χ2 = 1.896, P = 0.169	χ2 = 2.911, P = 0.088	χ2 = 3.539, P = 0.060
**9°**	χ2 = 3.995, P = 0.046	χ2 = 10.37, P = 0.001	χ2 = 6.841, P = 0.009
**10°**	χ2 = 3.343, P = 0.047	χ2 = 9.267, P = 0.002	χ2 = 5.660, P = 0.017

*POD: postoperative day.

## Discussion

Since Bazaz et al [8] prospectively used the dysphagia scoring system to assess postoperative dysphagia in 2002, the Bazaz dysphagia scale became the most popular scale for dysphagia evaluation. It defined dysphagia as none, mild, moderate and severe, depending on patients' symptoms with solid and liquid foods. However, this scoring system was simple and the incidence of dysphagia assessed by it may not reliable. Dysphagia Disability Index (DDI) is another popular scale which was used to evaluate the dysphagia after ACSS. It includes 25-item questionnaire to assess patients’ opinions of functional, emotional and physical dysphagia effects [9]. However, this scale is complicated and costs much time for patients to answer the questions. Recently, Swallowing- Quality of Life (SWAL-QOL) questionnaire is developed for dysphagia measurement [10, 11]. This questionnaire is a validated patient-based measure of dysphagia and requires patients to answer more than 60 questions at each postoperative visit, which is also not convenient for patients to use. In addition, some of the questions in SWAL-QOL scale are less applicable in spinal surgical study [12]. Therefore, a concise and explicit scoring system for dysphagia evaluation is necessary.

In the present study, we designed a new scoring system for dysphasia assessment. Dysphagia was defined as pharyngeal pain and foreign body sensation when swallowing solids or liquids in the new scoring system. And the scale was divided into five grades, which was easy and convenient for patients to understand. Moreover, it is more detail than Bazaz scoring system, because we added pharyngeal pain evaluation to the system, which will be more reliable on identifying dysphagia after ACSS. In the present study, the self-designed scoring system was compared with Bazaz scoring system. The results indicated all the correlation coefficients were greater than 0.65 (*P* < 0.001), which meant the two scoring system had a good correlation.

The reported incidence of dysphagia is widely variable due to the different measurements of dysphagia [13]. Rihn et al [14] found that the incidence of dysphagia at two week after ACSS was as high as 71%. Bazaz et al [8] followed up a series of 249 consecutive patients undergoing ACSS for 12 months, and found that the dysphagia incidences were 50.2%, 32.2%, 17.8% and 12.5% at 1, 2, 6, and 12 months, respectively. Kalb et al [9] reviewed the medical records of 249 patients who underwent ACSS, and defected that presences of dysphagia at 6 weeks, 3 months and 6 months were 88.8%, 29.6%, and 7.4%, respectively. However, most of previous studies just reported the incidence of long-term dysphagia, few studies focused on the immediate presence of it. Moreover, the rate of dysphagia after ACSS was significantly under-reported if it was analyzed based on the medical records [15]. In the current study, we used the new dysphagia scoring system to measure the immediate incidences of dysphagia after ACSS. And we conducted the survey face to face with patients, in order to decrease the rate of deviation. Based on the results, the incidences of dysphagia from the first to the fifth day after surgery were 87.5%, 79.81%, 62.14%, 50% and 44.23%, which were a litter higher than those reported in previous studies.

Several studies have reported the risk factors associated with dysphagia after ACSS. Lee et al [16] carried out a study with 2 years follow up, and found that gender, revision surgeries, and multilevel surgeries were the risk factors for long-term dysphagia after ACSS. Kalb et al [9] identified that multilevel procedures (especially involvement of C4-5 and C5-6) and age were the risk factors of dysphagia. Olsson et al [17] reported that the risk factors for long-term dysphagia were smoke and revision surgery. Different from previous studies, we found that operative time, BMI and dC2-C7 angle were the risk factor for early dysphagia in the present study.

The operative time and BMI were identified closely related to the incidences of early dysphagia from the first to the second day postoperatively. As you know, if the esophagus and prevertebral soft tissue were retracted for a long time during the surgery, they would be injured and resulted in severe swelling [18]. The swelling of esophagus and prevertebral soft tissue finally led to patient dysphagia. For the BMI, patient with a large BMI had a thick prevertebral soft tissue, which made it difficult to expose the surgical levels of the cervical spine, and finally increased the operative time. Based on the analysis, the dC2-C7 angle significantly associated with early dysphagia from the third to the fifth day. Tian et al [19] also found that the dC2-C7 angle was the risk factor of postoperative dysphagia, and claimed that when the dC2-C7 angle was greater than 5°, the chance of developing dysphagia was significantly greater. However, different from the results of Tian et al, we detected when the dC2-C7 angle was greater than 9°, the incidence of dysphagia was significantly increased.

Also, there are several limitations in this study. Firstly, the sample size of this study was small, and the dysphagia was only measured on the first to fifth day after surgery. The evidences for the accuracy and efficacy of the new scoring system on dysphagia assessment were not high enough. A randomized control studies with a larger sample size and a long-term follow-up is necessary to further determine the outcomes. Secondly, the dysphagia was reported just basing on the subjective feeling of patients, which may not be completely reliable. Thirdly, the sensitivity and specificity of the new scoring system were not analyzed in the present study. Advance study for the new scoring system will be helpful for the understanding and application of it.

In conclusion, we designed a new scoring system to evaluate early dysphagia after ACSS. And it is simple and convenient for patients to use. According to the new scoring system, the incidences of early dysphagia in patients were 87.5%, 79.81%, 62.14%, 50% and 44.23% on the first five days after surgery. The possible risk factors related to postoperative dysphagia were operative time, BMI and the dC2-C7 angle. Therefore, reducing operative time and decreasing the injury to the prevertebral soft tissue is helpful to decrease the incidence of dysphagia after ACSS. In addition, the dC2-C7 angle should be considered when performing the surgery.
